# Genetics of Hypercholesterolemia: Comparison Between Familial Hypercholesterolemia and Hypercholesterolemia Nonrelated to LDL Receptor

**DOI:** 10.3389/fgene.2020.554931

**Published:** 2020-12-03

**Authors:** Estíbaliz Jarauta, Ana Ma Bea-Sanz, Victoria Marco-Benedi, Itziar Lamiquiz-Moneo

**Affiliations:** ^1^Hospital Universitario Miguel Servet, Instituto de Investigacion Sanitaria Aragon (IIS Aragn), Zaragoza, Spain; ^2^Centro de Investigación Biomédica en Red Enfermedades Cardiovasculares (CIBERCV), Instituto de Salud Carlos III, Madrid, Spain; ^3^Department of Medicine, Psychiatry a Dermatology, Universidad de Zaragoza, Zaragoza, Spain

**Keywords:** primary hypercholesterolemia, familial hypercholesterolemia, polygenic hypercholesterolemia, cardiovascular disease, LDL-cholesterol, atherosclerosis

## Abstract

Severe hypercholesterolemia (HC) is defined as an elevation of total cholesterol (TC) due to the increase in LDL cholesterol (LDL-C) >95th percentile or 190 mg/dl. The high values of LDL-C, especially when it is maintained over time, is considered a risk factor for the development of atherosclerotic cardiovascular disease (ASCVD), mostly expressed as ischemic heart disease (IHD). One of the best characterized forms of severe HC, familial hypercholesterolemia (FH), is caused by the presence of a major variant in one gene (*LDLR, APOB, PCSK9*, or *ApoE*), with an autosomal codominant pattern of inheritance, causing an extreme elevation of LDL-C and early IHD. Nevertheless, an important proportion of serious HC cases, denominated polygenic hypercholesterolemia (PH), may be attributed to the small additive effect of a number of single nucleotide variants (SNVs), located along the whole genome. The diagnosis, prevalence, and cardiovascular risk associated with PH has not been fully established at the moment. Cascade screening to detect a specific genetic defect is advised in all first- and second-degree relatives of subjects with FH. Conversely, in the rest of cases of HC, it is only advised to screen high values of LDL-C in first-degree relatives since there is not a consensus for the genetic diagnosis of PH. FH is associated with the highest cardiovascular risk, followed by PH and other forms of HC. Early detection and initiation of high-intensity lipid-lowering treatment is proposed in all subjects with severe HC for the primary prevention of ASCVD, with an objective of LDL-C <100 mg/dl or a decrease of at least 50%. A more aggressive reduction in LDL-C is necessary in HC subjects who associate personal history of ASCVD or other cardiovascular risk factors.

## Introduction

Severe primary hypercholesterolemia (HC) is a disorder of lipid metabolism, clinically characterized by an elevation of LDL cholesterol (LDL-C) >190 mg/dl and/or total cholesterol (TC) >95th percentile or >300 mg/dl, with normal values of triglycerides (TGs). Despite the great efforts and health plans carried out to improve the detection and clinical management of HC subjects, that population still remains underdiagnosed and undertreated (Nordestgaard et al., 2013; Representatives of the Global Familial Hypercholesterolemia Community et al., 2020). Classically, severe HC constitutes an inherited trait, frequently associated with high cardiovascular risk due to lifelong exposure to elevated cholesterol levels, causing ischemic heart disease (IHD) as the main clinical manifestation (Civeira and International Panel on Management of Familial Hypercholesterolemia, 2004; Mach et al., 2020). One of the best known cause of HC is familial hypercholesterolemia (FH)# 143890, a genetic disorder with an autosomal codominant inheritance pattern, due to a monogenic defect in LDL receptor (*LDLR*), apolipoprotein B (*APOB*), Proprotein convertase subtilisin/kexin type 9 (*PCSK9*), or apolipoprotein E (*APOE*) genes, involved in the LDL receptor endocytic and recycling pathways.

However, a deeper knowledge about genomics has made it possible to detect genetic variations related to a specific trait. One of the most extended methods, genome-wide association studies (GWAS), allows to detect hundreds of single variations in a nucleotide (SNVs) in a unique subject, located throughout the genome. Some of these SNVs may be associated with differences in LDL-C serum values and IHD. Afterwards, it makes possible to compare the same unbiased genome screens of unrelated individuals and appropriately matched controls. When a number of these SNVs cluster in the same subject, it has been set out as a cause of primary HC (Teslovich et al., 2010), being labeled as polygenic hypercholesterolemia (PH) (Talmud et al., 2013). Nonetheless, there still remains a proportion of HC subjects in whom no significant increase is detected on SNVs related to LDL-C with respect to the general population. Hitherto, the prevalence, diagnosis, and clinical management of subjects with PH have not been set up. The aim of this review was to describe the clinical profile, inheritance pattern, and treatment of subjects with PH, as well as the main difference between monogenic and non-monogenic origen of HC.

## Primary Hypercholesterolemia as a Driving Factor for the Development of Cardiovascular Disease

Classically, severe HC has been established as a causal risk factor of atherosclerotic cardiovascular disease (ASCVD) (Ross, 1999; Nordestgaard et al., 2013). However, a positive correlation between TC and ASCVD has been observed in men and women, as a continuous variable from TC values >180 mg/dl, whereas a very low incidence of ASCVD has been observed in those with TC below that value (Stamler et al., 1986; Nagasawa et al., 2012; Kwon et al., 2019; Maihofer et al., 2020). Similarly, the treatment with hypolipemiant drugs maintained over the years has demonstrated a significant decrease in IHD and cardiovascular mortality in subjects with primary HC (Besseling et al., 2014; Humphries et al., 2018; Perez-Calahorra et al., 2019).

**Figure 1:**
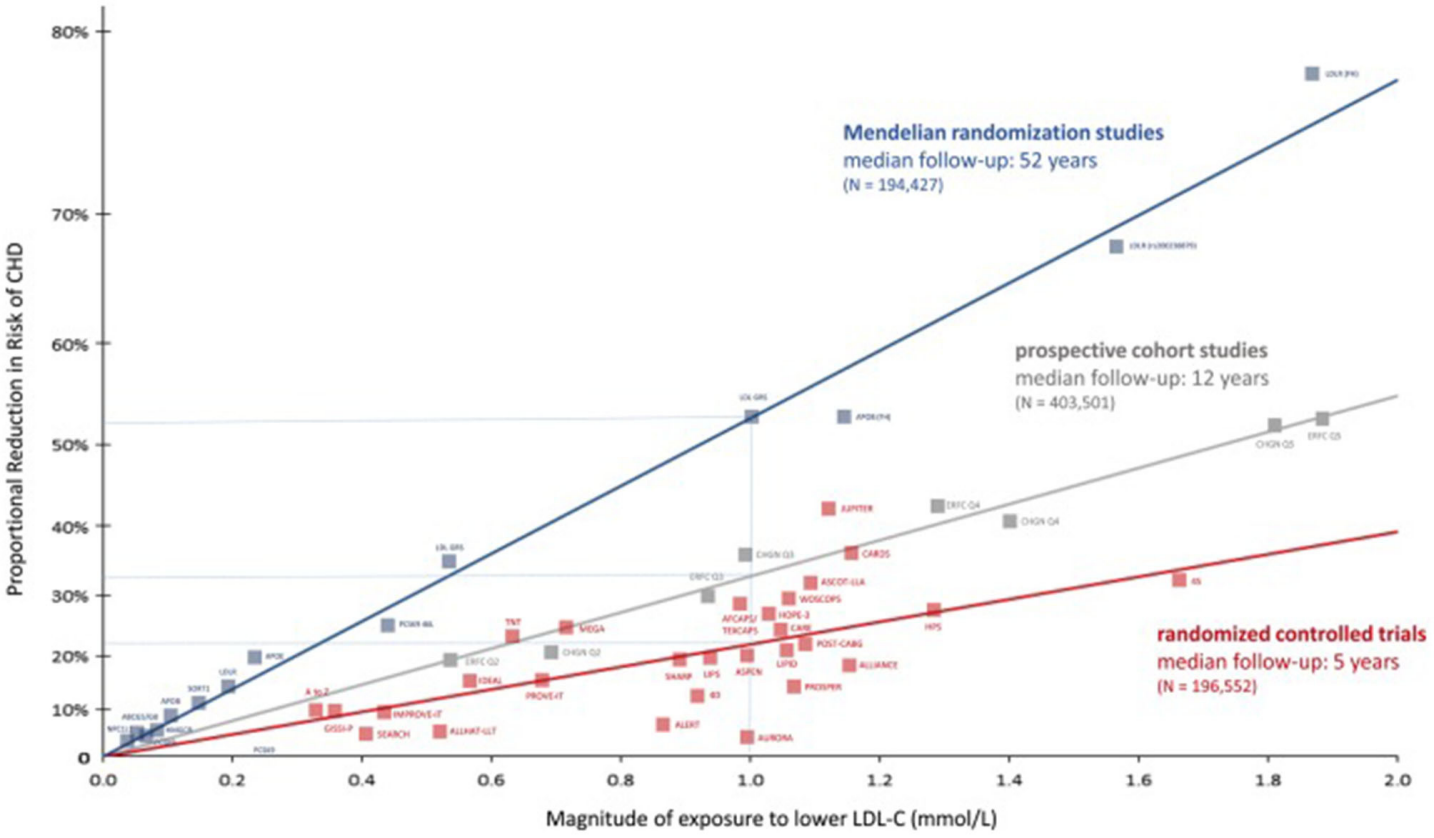
Reproduced with permission from Ference et al. (2017). Log-linear association per unit change in low-density lipoprotein cholesterol (LDL-C) and the risk of cardiovascular disease as reported in meta-analyses of Mendelian randomization studies, prospective epidemiological cohort studies, and randomized clinical trials.

Recent meta-analyses of prospective cohort studies, Mendelian randomization studies, and randomized controlled trials, including more than 2 million of participants, allowed to establish a log-linear, dose-dependent association between TC, non-HDL cholesterol (non-HDL-C) and LDL-C, and the risk of IHD and mortality (Figure 1). All of them are surrogate markers of apolipoprotein B (ApoB), an apoprotein that transports the majority of cholesterol in the blood stream. From among them all, LDL-C is the best correlated to ApoB, since it contains up to 90% of all apoB detected, while in non-HDL-C and TC, a bigger amount of TG and other lipoparticles is included (Ference et al., 2017).

All these studies offered the possibility of establishing a temporal association between LDL-C exposure and IHD. However, Mendelian randomization studies go a step further introducing a lifelong randomization scheme by the genetic variants associated with serum LDL-C values (Ference et al., 2012; Holmes et al., 2015) (Figure 1). The GWAS and custom genotyping arrays, carried out in populations from European, American, and African ancestry, have allowed to detect a number of SNV linked up with the catabolism and overproduction of LDL particles as well as the prevalence of IHD (Willer et al., 2013). Each of these variants is approximately inherited randomly at the time of conception in a process referred as “Mendelian randomization” (Ference, 2015). If there is no association with any other variant in other gene-modifying LDL-C, that genetic variant should provide and estimation of the effect on LDL-C levels. It is possible to establish a relation between that genetic variant and the risk of ASCVD, analogously to a long-term clinical trial. These studies have demonstrated that only variants in genes that modify LDL-C, but not in another traits of lipid profile, are associated with a lower risk of IHD. Finally, randomized control trials show the reduction in ASCVD incidence by reducing LDL-C. In a classical meta-analysis including 170,000 subjects, a decrease of 1 mmol (38 mg/dl) of LDL-C by statins was associated with a 22% reduction in the risk of major cardiovascular events over a median of 5 years of treatment. The magnitude of this reduction was independent of baseline LDL-C and any type of hypolipemiant drug used, and its results are remarkably consistent in different subgroups of patients studied [(Cholesterol Treatment Trialists' (CTT) Collaborators et al., 2008; Wang et al., 2020)] (Figure 1).

The comparison of data from these three sources has enabled to draw a figure relating the magnitude of LDL-c exposition and the proportional reduction in risk of coronary heart disease observed in the three types of studies. Interestingly, with the same magnitude of lowering LDL-C, the biggest effect was observed in Mendelian randomized studies, emphasizing not only the importance of the serum values of LDL-C but also the time of exposure, expressed as the LDL burden for atherosclerosis (Figure 1) (Ference et al., 2017). High serum concentration of LDL particles maintained over the time implies a high burden of ApoB, which can cross the endothelial barrier and interact with other wall components, leading to the thickening of the arterial walls and the development of atherosclerotic plaques. Some of the mechanisms implied as vascular inflammation is related to the pathological internalization of apoB-containing particles by macrophages, especially when LDL particles become oxidized or are otherwise modified (Borén et al., 2020). Those damaged plaques may evolve to be broken, especially when it contains a necrotic core and macrophage accumulation full of LDL particles (Ross, 1999). Recent findings have correlated the descent in LDL-C with a decrease in atherosclerotic plaque volume and ASCVD (Forbes et al., 2016).

## Genetics of Primary Hypercholesterolemia: From Major Variants in a Unique Gen to the Additive Effects of Single Nucleotide Variants in Multiple Genes

A new approach to genetic diagnosis of primary HC has been developed: from searching for new monogenic variations with large effect on disease status, to the additive effect of several small variants with little pathogenic effect on several genes related to lipids metabolism in the same subject (Berberich and Hegele, 2019). The traditional definition of severe HC corresponds to a monogenic disease in which one copy of a variant allele produces the disease phenotype. The first cases of HC associated with a familial pattern, tendinous xanthomas (TX), and early mortality were reported in the 1930s by Carl Müller, based on the findings of 17 families with xanthomata and early IHD in Norway (Ose, 2002). It was not until the 1970s that the identification of a variant of *LDLR* causing lack of affinity of LDL particle to the LDL receptor enabled Goldstein and Brown to establish the pathogenesis of FH (Goldstein and Brown, 1974). Until now, more than 2,000 causative variants of FH have been described, the majority (80%) located in *LDLR* and the rest in *APOB* and *PCSK9* genes. The effects of that allelic variants encompass any of the stages of receptor-mediated endocytosis of LDL particles. The effect of large *LDLR* variants can be sorted out in two categories: no protein synthesis or synthesis of a totally non-functional receptor, whereas large *APOB* variants affect the receptor-binding domain of ApoB. Finally, *PCSK9* variants with a gain of function increase the LDL-receptor recycling process, decreasing its half-life and its availability in the cell surface. Since all of them correspond to pathogenic variants that are expressed with autosomal codominant inheritance pattern, they have been grouped as causes of FH (Awan et al., 2013; Cenarro et al., 2016; Chora et al., 2018). Recently, the p.(Leu167) polymorphism in *APOE* gene has been associated with *LDLR* downregulation, raising LDL plasma levels, with clear familial segregation and the presence of TX, accounting for 3.1% subjects with PH and negative variants *in LDLR, APOB*, and *PCSK9* genes (Awan et al., 2013; Cenarro et al., 2016). In an attempt to find other new genes causing PH phenotype, a variant in signal transducer adaptor family member 1 (*STAP1*) gene causing FH phenotype was described in a large Dutch family (Fouchier et al., 2014). However, its role in FH seems to have been discarded recently (Hegele et al., 2020; Lamiquiz-Moneo et al., 2020).

Regarding PH, the first definition was provided by Talmud et al. (2013). From 27 SNVs related to LDL-C serum values, they built a score by the 12 more predictive of PH, observing a maximum difference of 44 mg/dL in LDL-C concentration between subjects in the highest and the lowest decile. In the same study, 52% of HC subjects non-carriers of large variants causing FH had a score within deciles 7–10 of SNVs distribution. Similar findings were reproduced in populations from Wales and Belgium (Talmud et al., 2013).

Afterwards, the same authors simplify the diagnosis of PH, removing the least frequent or least predictive SNVs, leaving only 6 from the initial 12, obtaining similar yields in the diagnosis of PH. These polymorphisms were located in genes involved in different pathways of LDL particles metabolism such as *APOB, LDLR*, and *APOE* genes. Others, as *ABCG5/8* (ATP-binding cassette, subfamily G, member 5/8), modulate cholesterol production by sterols hyperabsorption (Baila-Rueda et al., 2016); meanwhile, *CELSR2* (cadherin, EGF LAG 7-pass G-type receptor 2) is related to cells signaling, although its function with respect to LDL-C metabolism is unknown (Paththinige et al., 2017). In a group of 1,158 probands with HC and a family history of premature ASCVD, the polygenic score built with those SNVs was able to diagnose 36% of subjects with PH when the cutoff value was 75th percentile in SNVs distribution. These results were reproduced in seven cohorts of HC subjects from different countries (Futema et al., 2015). However, when we tried to replicate the same experiment in our population, no differences in its prevalence between HC and normolipemic members of the same family were observed, explaining the 6.9% of LDL-C value in HC subjects (Lamiquiz-Moneo et al., 2017). Another large study carried out in 5,415 subjects who belonged to the general population included nine SNVs from genes related to LDL-C and HDL-C metabolism (*LDLR, APOB, APOE*, and *ABCG5/8* among others), observing a maximum difference of 72 mg/dl between the homozygote classes of SNVs. Despite the fact that this score was an independent risk factor for ASCVD, the genotype did not improve ASCVD prediction with respect to classical cardiovascular risk factors (Kathiresan et al., 2008).

More recently, a deeper coverage of whole genome sequencing analyses by next-generation sequency technology has enabled to detect monogenic variants, in addition to hundreds of SNVs, gene copy numbers, and genomic rearrangements from the various types of DNA-sequencing and microarray data, related to lipid metabolism and/or ASCVD (Natarajan et al., 2018). Nevertheless, no new variants related to LDL-C have been detected. These SNVs have been located in genes encoding structural components of lipoproteins, lipoprotein receptors and related proteins, enzymes, lipid transporters, lipid transfer proteins, and activators or inhibitors of some protein function and gene transcription. However, some of them are within or in the vicinity of genes that are not known to be involved in lipid metabolism. Besides, over 90% of these SNVs are located outside the coding regions, hence will be missed in routine exome-sequencing techniques (Paththinige et al., 2017). Each polymorphism is associated with small but reproducible increase in LDL-C levels, explaining the impact of each one between 0.1 and 2.5% of LDL-C serum values (Kathiresan et al., 2008; Willer et al., 2013; Lamiquiz-Moneo et al., 2017; Trinder et al., 2020).

**Figure 2:**
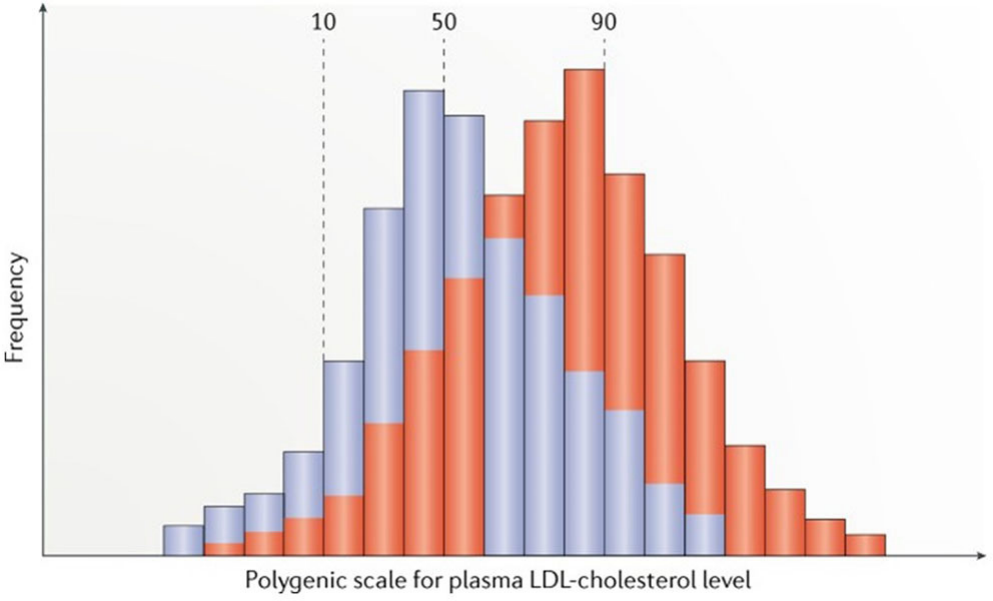
Reproduced with permission from Berberich and Hegele (2019). Scheme of distributions for polygenic risk scores for LDL-cholesterol (LDL-C) levels in the general normolipidemic population (blue) and in clinically ascertained patients with suspected familial hypercholesterolemia (FH) but no monogenic variant in genes causing FH (red). Scores are calculated from single nucleotide polymorphism genotypes, by simply tallying trait-raising alleles, or scores can be further weighted according to effect sizes for the alleles reported in genome-wide association studies.

Because polygenic LDL-C *loci* are scattered throughout the genome and segregate independently during meiosis, most individuals have an overall balance between LDL-C-raising and LDL-C-lowering alleles. Rare individuals at the high extreme of polygenic scores have inherited a preponderance of LDL-C-raising alleles (Figure 2) (Berberich and Hegele, 2019) and frequently clustered and inherited in the same family by mechanisms not described hitherto (Table 1) (Jarauta et al., 2016; Berberich and Hegele, 2019). Furthermore, these SNVs seem to contribute to the severity of HC and increased IHD in some FH subjects that exhibit higher LDL-C levels with respect to FH without a polygenic inheritance (Talmud et al., 2013; Ghaleb et al., 2018; Trinder et al., 2019). Nowadays, the diagnosis of PH varies depending on the number and type of SNVs included and the percentage cutoff point chosen for the diagnosis, without any standard definition for PH accepted at the moment (Table 2). Thus, the identification of a specific genetic pathological variant is not a necessary condition for the diagnosis of a genetic HC (Berberich and Hegele, 2019).

**Table 1:** Heritage pattern analysis and heritability by family-based association test in HC families non-related to FH (Jarauta et al., 2016).

**Dependent Variable**	**Dominant model (number of informative trios = 55)**			**Recessive model (number of informative trios = 23)**			**Covariates**
	***P*-value (FBAT)**	**Power (FBAT)**	**Heritability**	***P*-value (FBAT)**	**Power (FBAT)**	**Heritability**	
Total cholesterol	3.92E-14	0.999	0.389^*^	0.001	0.934	0.059^*^	Sex, age, BMI, *APOE*
Triglycerides	0.114	0.250	0.028	3.79E-06	0.999	0.178^*^	Sex, age, BMI, *APOE*
HDL cholesterol	0.600	0.855	0.118	0.047	0.124	−0.012^*^	Sex, age, LDLc, BMI, *APOE*
LDL cholesterol	2.80E-14	0.999	0.322^*^	0.005	0.674	0.035^*^	Sex, age, HDLc, BMI, *APOE*

*BMI, body mass index; FBAT, family-based association tests; HDL, high-density lipoprotein; LDL, low-density lipoprotein; HDLc, high-density lipoprotein cholesterol; LDLc, low-density lipoprotein cholesterol; non-FH-GH, non-familial hypercholesterolemia-genetic hypercholesterolemia*.

* *Statistically significant model. Variables with significant univariate association with the lipid profile were included as covariates*.

**Table 2:** Principal studies including data about prevalence of familial hypercholesterolemia and polygenic hypercholesterolemia.

**Study first author, year**	***N***	**Clinical characteristics**	**Time follow-up (years)**	**Number and genes with SNVs included for diagnosis of PH**	**Prevalence of PH (%), % of score**	**Prevalence of FH (%)**	**ASCVD risk FH**	**ASCVD risk associated to PH**
Kathiresan et al. (2008)	5,414	Cardiovascular cohort Malmö Diet and Cancer Study	10.6	*9 (APOB, PCSK9 LDLR*; *CETP LIPC LPL, APOE, HMGCR, ABCA1)*	26.2 (>fourth quartile)	NA	NA	1,63 hazard ratio
Willer et al. (2013)	1,158	PrH in proband plus family history of premature myocardial infarction	NA	*6 (CELSR2, APOB, ABCG5/8 LDLR and APOE)*	36 (>fourth quartile)	351 (33,3)	NA	NA
Khera et al. (2016)	26,025	5,540 coronary artery disease	NA	NA	NA	1.9	22	6 (HC subjects non-FH)
Khera et al. (2018)	2,081	Early onset of myocardial infarction	NA	^*^6,6 × 10^6^	17.3 (>95th decile)	1.7 (*LDLR truncation, frameshift, splicing)*	3.8	3.7
Benn et al. (2012)	69,016	Danish community-based population	NA	NA	6.9 (definitive, probable or possible FH)	0.2	10.3	NA
Benn et al. (2016)	98,098	Copenhagen general population Study	36	NA	7.2	0.46	5.3 *LDLR* carriers 1.8 *APOB* carriers	NA
Natarajan et al. (2018)	16,324	From 4 ancestries^**^	NA	2 × 10^6^-SNV LDL-C polygenic score	23% of HC (>95th decile)	2% of HC	NA	NA
Patel et al. (2019)	1.18 × 10^6^	Geisinger Health System patients	NA	NA	13.7	0.15 (definitive FH)	NA	1.52
Trinder et al. (2019)	626	British Columbia FH patients according to DLCN, <55 yr. and myocardial infarction	7.2	28	21.4 (>80th percentile)	43.9 (frameshift novel and no sense in LDLR and APOB mutations; LDLR variants <1% and pathogenic)	1.97	1.39
Trinder et al. (2020)	48,718	UK biobank	7.2	223	4.9 (>95th percentile)	0.57	1.93	1.29

* *Variants drawn were related to LDL-C and myocardial infarction trait*.

** *Subjects with lipid profile available from Framingham Heart Study (FHS), Old Order Amish (OOA), Jackson Heart Study (JHS), Multi-Ethnic Study of Atherosclerosis (MESA), FINRISK Study (FIN), and Estonian Biobank (EST)*.

*In case that PH score study was not available, data referred as PH express the prevalence of primary hypercholesterolemia (LDL-C >95th percentile) non-related to FH. Type of event evaluated were myocardial infarction, ischemic stroke, and death from coronary heart disease. “Definite,” “probable,” or “possible FH” was defined by the Dutch Lipid Clinic network score (>8, 6–8, 5–7, respectively). PH, polygenic hypercholesterolemia; FH, familial hypercholesterolemia; ASCVD, atherosclerotic cardiovascular disease; SNV, single nucleotide variation; NA, non-available; NS, non-significant*.

*APOB, apolipoprotein B; APOE, apolipoprotein E; CELSR2, cadherin; CETP, cholesteryl ester transfer protein; EGF LAG 7-pass G-type receptor 2; HMGCR, 3-hydroxy-3-methylglutaryl-coenzyme A reductase; LDLR, low-density lipoprotein receptor; LIPC, hepatic lipase; LPL, lipoprotein lipase; PCSK9, proprotein convertase subtilisin/kexin type 9*.

## Are Polygenic Hypercholesterolemia and Familial Hypercholesterolemia Clinically the Same or Different? Comparison of Criteria for Clinical Diagnosis, Lipid Profile, and Cardiovascular Disease

Recent data delivered a higher prevalence of FH than previously documented, with a general pooled estimation in 1:250 (0.4%) (Benn et al., 2016; Akioyamen et al., 2017) accounting about 1.7–5.6% of subjects with severe HC (Benn et al., 2012; Khera et al., 2016; Trinder et al., 2020). On the contrary, other forms of HC comprise a larger proportion of individuals in both population-based approach studies or large samples from different genetic backgrounds: 13.7% of 1.18 million of adults in the United States (Patel et al., 2019), about 10% of 48,781 participants in the UK Biobank cohort study (Trinder et al., 2020), up to 7% of 67,019 subjects from the Danish general population study (Benn et al., 2012) and 6.8% of 20,485 ASCVD-free subjects belonging to 12 cohorts from different countries (Khera et al., 2016) (Table 2).

Conversely, when starting from ASCVD cases, the clinical diagnosis of FH increases significantly, being reported in 6.7% patients suffering from first myocardial infarction (Mortensen et al., 2016). Besides, in case of premature myocardial infarction, differences in FH and PH prevalence disappeared. In a study including 626 HC patients younger than 55 years old with a personal history of IHD, FH prevalence was doubled than PH (43.9 vs. 21.4%), with the highest cardiovascular risk for FH subjects with an added polygenic trait for LDL-C. In addition, PH was not associated with an increased cardiovascular risk with respect to those HC subjects with no associated genetic trait for LDL-C (Trinder et al., 2019). On the contrary, in a study including 2,041 subjects with premature onset of myocardial infarction, prevalence of PH was 10 times higher than those with FH, showing a similar risk for IHD between PH and FH subjects. However, in that case, the diagnosis of PH was not based on LDL-C serum values, rather on the 95th percentile of 6.6 million of common DNA variants associated with myocardial infarction detected by deep coverage whole exome sequencing (Khera et al., 2018) (Table 2).

The main clinical characteristics of FH as the extreme elevation of LDL-C, premature ASCVD, the presence of TX, and family history of severe HC and premature ASCVD have been collected in several scores as predictors of FH, since genetic diagnosis is not affordable in all cases (Representatives of the Global Familial Hypercholesterolemia Community et al., 2020). The most popular, the Dutch Lipid Clinic Network (DLCN) score, classifies subjects as “unlikely FH” (score < 3), “probable FH” (score = 3–5), “possible FH” (score = 6–8), and “definite FH” (score > 8) (Table 3). Quite similarly, the Simon Broome Score labels subjects as “definite FH” if LDL-C is above 190 mg/dl and they have TX and “possible FH” for those with familial history of premature IHD besides the elevation of LDL-C. In both of them, a higher score or the presence of TX increased significantly the probability of FH diagnosis (Civeira et al., 2008b; Palacios et al., 2012; Benn et al., 2016; Trinder et al., 2019).

**Table 3:** Dutch Lipid Clinic Network score criteria for diagnosis of heterozygous familial hypercholesterolemia: odds ratio of every item for the diagnosis of FH.

	**Points**	**Odds ratio for FH (Civeira et al., 2008b) *N* = 825**	**Odds ratio for FH, (Benn et al., 2016) Danish General population *N* = 98,098**	**Odds ratio for FH, (Palacios et al., 2012) Hypercholesterolemic population *N* = 5,430**	**Odds ratio for FH %(Trinder et al., 2019) HC subjects <55 yr + Myocardial infarction**
-First degree relative with known premature coronary heart disease or	1	NA	1.3(0.9–2.0)^*^	NA	NA
-First degree relative with known LDL-C >95th percentile by age and gender for country	1	NA	5.2(3.8–7.1)^*^	NA	NA
-First degree relative with tendon xanthoma and/or corneal arcus or	2	7.8	NA	NA	NA
-Child(ren) < 18 years with LDL-C > 96th percentile by age and gender for country	2	NA	NA	NA	NA
-Subject has premature coronary heart disease	2	NA	3.2(1.8–5.6)^*^	NA	NA
-Subject has premature cerebral or peripheral vascular disease	1	NA	0.8(0.3–1.9)^*^	NA	NA
Tendon Xanthoma	6	3.7	NA	NA	NA
Corneal arcus in a person <45 years	4	2.6	NA	NA	NA
LDL-C> 325 mg/dL	8	NA	138 (60–318)^†^	NA	NA
LDL-C> 251–325 mg/dL	5	NA	53(35–80)^†^	NA	NA
LDL-C>191–250 mg/dL	3	NA	53(35–80)^†^	Na	NA
LDL-C> 155–190 mg/dL	1	NA	25(19–34)^†^	NA	NA
Definite FH: DLCN >8		NA	24^$^	53,9^$^	74.3
Possible FH: DLCN 6–8		NA	6^$^	30,7^$^	37.4
Probable FH: DLCN 3–5			1,2^$^	23,9^$^	11.8
Unlike FH: DLCN <3			0,07^$^	16,4^$^	NA

*Table built with data from Civeira et al. (2008b), Palacios et al. (2012), Benn et al. (2016), and Trinder et al. (2019)*.

*LDL-C denotes low density cholesterol, DLCN denotes Dutch Lipid Clinic Network. Premature coronary heart disease was considered <55 years, men; <60 years, women*.

* *Odds ratios for each criterion are risk of carrying a variant in individuals fulfilling the specific criteria versus those not fulfilling the same criteria used as reference group*.

† *Odds ratios in groups by low-density lipoprotein (LDL)-cholesterol levels are risk of carrying a variant in genes causing FH in individuals with an LDL-cholesterol level above the threshold compared those below*.

$ *Percentage of subjects with pathogenic variant causing FH according to DLCN category*.

TX constitutes nearly a pathognomonic sign of FH, increasing the odds ratio for the diagnosis of FH (Table 3). Nonetheless, it might be detected in some cases of sytosterolemia, an autosomal recessive form of HC caused by defects on *ABCG5* and *ABCG8* genes, which increases plasma sterols absorption (Bastida et al., 2019). TX are composed of an extravascular deposit of LDL-C and non-cholesterol sterols (Baila-Rueda et al., 2018), resembling the lipid composition of vascular atheromatous plaques (Vermeer et al., 1982). The presence of TX has been associated with early IHD independently of the genetic variant causing FH (Civeira et al., 2005). However, the prevalence of TX in FH subjects is only about 40% (Civeira et al., 2008b), increasing up to 68% when sonographic evaluation is used for the diagnosis (Junyent et al., 2005). A lower prevalence of TX has been observed in the last decades due to long-term intensive lipid-lowering therapy and the early diagnosis of FH (Benn et al., 2012; Bea et al., 2017; Berberich and Hegele, 2019). Consequently, the absence of TX defines most of the subjects with HC as “probable FH” or “possible FH” (Patel et al., 2019), decreasing the sensitivity of DLCN and Simon Broome scores for the diagnosis of FH (Cuchel et al., 2014; Trinder et al., 2020).

**Figure 3:**
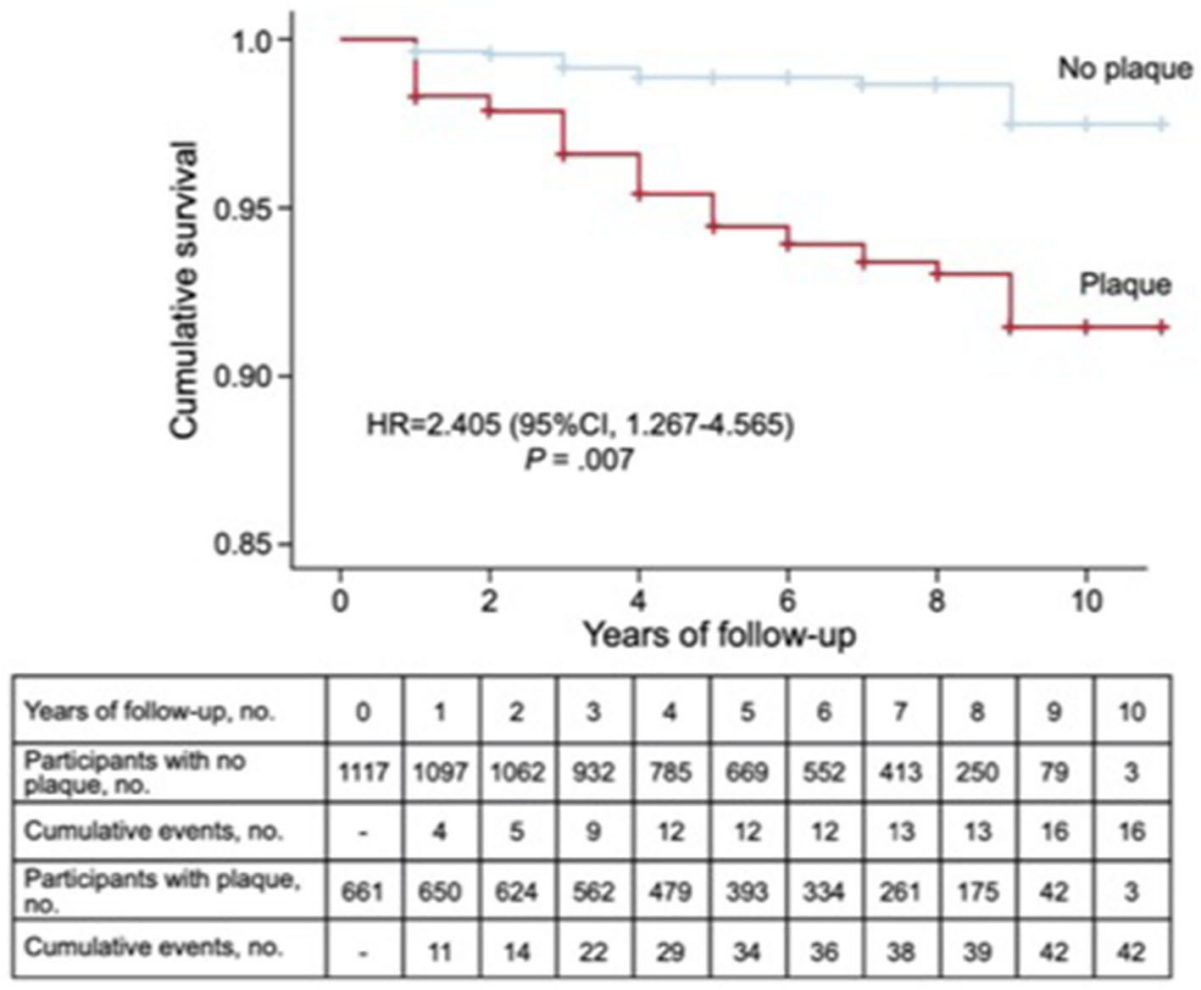
Prevalence of subclinical atherosclerosis measured by carotid plaques detected by ultrasound and incidence of ASCVD in a population of 1,771 subjects with primary hypercholesterolemia. Kaplan–Meier cumulative survival curves for patients with and without arteriosclerotic plaque in carotid arteries. 95% CI, 95% confidence interval; HR, hazard ratio adjusted by history of cardiovascular disease, presence of carotid plaque, age, and sex.

The choice of a high value of LDL-C, above 99th percentile (>250 mg/dl), and normal values of TG is highly sensitive but not too specific for the diagnosis of FH (Civeira and International Panel on Management of Familial Hypercholesterolemia, 2004; Benn et al., 2016; Trinder et al., 2019). In fact, a significant overlap between LDL-C values in FH and PH subjects has been observed (Figure 3) (Nordestgaard et al., 2013; Trinder et al., 2020). Besides, most population registers are based on lipid profile and personal history of ASCVD, whereas a familial history of HC or ASCVD is not available (Benn et al., 2016; Khera et al., 2016; Trinder et al., 2020). All these make more difficult to rule out the diagnosis of FH from a clinical perspective.

Despite all of the above, FH cannot be discarded in all cases when TGs are elevated. In 143 subjects with a clinical diagnosis of familial combined hyperlipidemia, 19.6% carried a variant in *LDLR* causing FH. This corresponds to non-diabetic subjects with lower waist circumference and higher LDL-C serum, without a significant difference in TG serum values (Civeira et al., 2008a). In the same vein, a recent study including 49 families collected from 49 probands with severe HC and TG <90th percentile ruled out a monogenic cause; up to 25% of family members with dyslipemia had serum values of TG above 90th percentile. In addition, a polygenic nature of the disease and the influence of environmental factors were observed, which are expressed by the lipid profile variability and the coexistence of different heritage patterns for TC, LDL-C, and TG in the same family (Table 1) (Jarauta et al., 2016).

IHD disease is the most frequent clinical expression of ASCVD in both FH and PH patients (Benn et al., 2016; Khera et al., 2018; Trinder et al., 2020). Nevertheless, cardiovascular risk may vary within subjects with severe HC. In patients clinically diagnosed as FH followed during the last 30 years, those with “definite FH” according to Simon Broome criteria showed 2.4-fold excess of coronary mortality, whereas the same excess risk was 1.7 in “probable FH.” The excess risk continued being higher in subjects with previous IHD and a genetic diagnosis of FH despite the decrease in cardiovascular risk in the general population and the rest of the HC population during the same period of follow-up. Surprisingly, in the same study, the reduction in mortality in women over three decades was less important than the one observed in men despite the same efficacy observed on lowering LDL-C in men and women treated with statins [(Cholesterol Treatment Trialists' (CTT) Collaborators et al., 2008)]. This fact raises the question of whether these “definite FH” women are being treated as rigorously as their male counterparts (Humphries et al., 2018).

Likewise, those subjects with PH present more frequently ASCVD in other locations as cerebrovascular or peripheral arteries and at older ages than FH. This fact has been related to the lower mortality and the higher prevalence of other cardiovascular risk factors observed in PH subjects (Benn et al., 2012; Perez-Calahorra et al., 2019). Other forms of HC out of the scope of this review, such as familial combined hyperlipidemia or hypercholesterolemia secondary to the elevation of lipoprotein (a), increase the risk of ASCVD with regard to the general population (Langsted et al., 2016; Berberich and Hegele, 2019; Luijten et al., 2019).

## Approach for the Screening and Management of Primary Hypercholesterolemia

**Figure 4:**
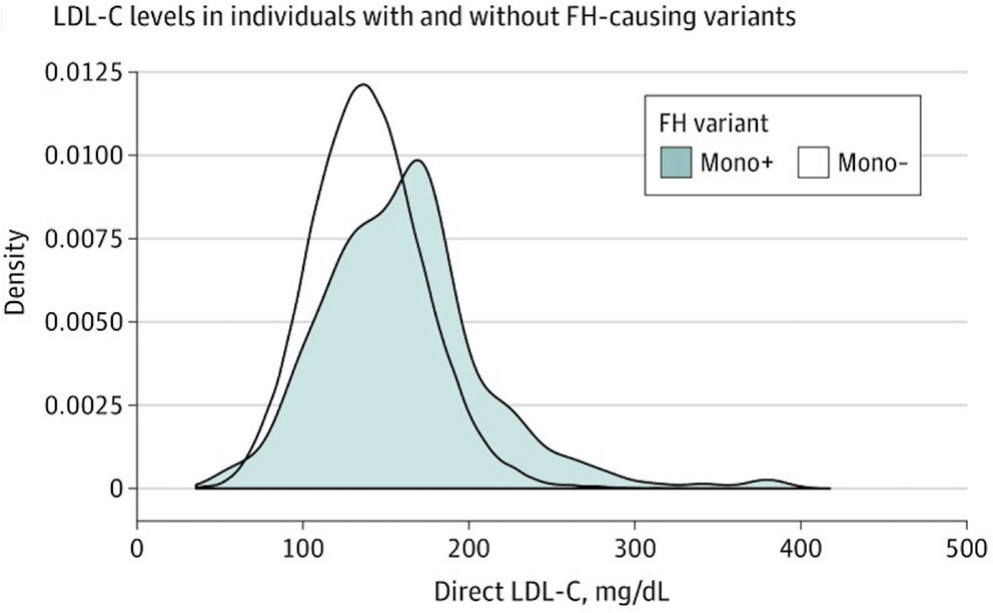
Reproduced with permission from Trinder et al. (2020). Distribution of LDL-C levels (to convert to millimoles per liter, multiply by 0.0259) at enrollment between individuals with an FH-associated variant (mono+) and those without an FH-associated variant (mono–).

The clinical significance of PH is as much as FH, since the prevalence of severe HC and polygenic origin of disease is more frequent in the general population (Khera et al., 2018; Berberich and Hegele, 2019; Trinder et al., 2020). Despite the fact that it may lead to misclassification bias for FH, the necessity of an inclusive strategy has been considered. On the basis of LDL-C serum values >90th percentile or >190 mg/dl in the adulthood, it is possible to identify most patients with higher risk for FH and ASCVD (Figure 4). The early diagnosis and treatment of severe HC should be considered a key point to prevent ASCVD in these subjects (Nordestgaard et al., 2013; Humphries et al., 2018; Perez-Calahorra et al., 2019). The higher IHD risk in FH population, especially at early ages, makes it necessary to carry out genetic diagnosis by cascade screening in patients clinically identified as “possible or definite FH” by DLCN score (Benn et al., 2012; Palacios et al., 2012; Humphries et al., 2018; Trinder et al., 2019). Nonetheless, a genetic diagnosis of PH is not available. In addition to the difference in the predictive SNVs depending on the study, its prevalence is not well-correlated to the presence of severe HC in all cases (Lamiquiz-Moneo et al., 2017).

**Figure 5:**
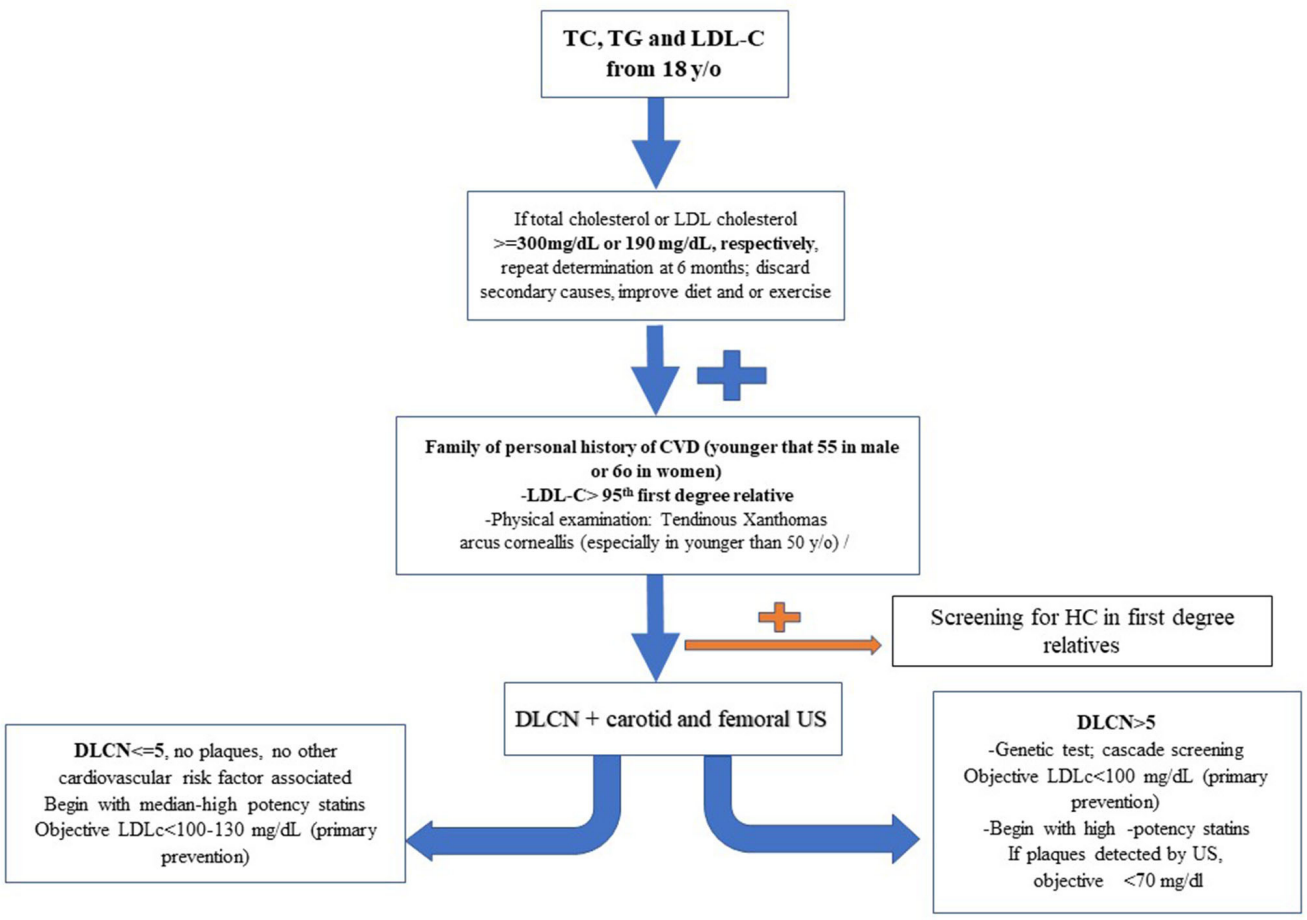
Scheme proposed about how to manage severe primary hypercholesterolemia.

The prevalence of subclinical atherosclerosis may be a useful method to detect those HC subjects whose ASCVD risk may be increased. However, only a few studies comparing subclinical atherosclerosis in FH and HC subjects are available so far. The Coronary Artery Calcium Score was a discriminative measurement of subclinical atherosclerosis for FH, showing higher atherosclerotic burden for FH than other HC patients (Sharifi et al., 2017). In a recent study, after a follow-up of 3.7 years, a 3.3-fold-higher risk of ASCVD for FH was observed when the calcium Agastont score was >100 with respect to those with a calcium Agastont score = 0 (Miname et al., 2019). Besides, the detection of atherosclerotic plaques in the carotid arteries by ultrasound increased 2.4-fold the risk of ASCVD in 1,778 subjects with HC, after a follow-up of 6 years (Bea et al., 2017) (Figure 5).

There is neither a case–control study to compare the effects of lipid-lowering drugs vs. placebo for the treatment of FH subjects nor any specific risk equations for ASCVD in this population (Nordestgaard et al., 2013). However, statins of moderate–high intensity, ezetimibe, and *PCSK9* monoclonal antibodies have shown a drop in the incidence of IHD and mortality in subjects with FH or severe HC (Versmissen et al., 2008; Karatasakis et al., 2017; Giugliano et al., 2018).

Clinical management of PH and FH in primary prevention does not differ from each other, since, until recently, the diagnosis of severe HC has been based on lipid profile and history of IHD. Consequently, there are few data for FH and PH separately. Nonetheless, a 10 times lower odds for CVD has been observed in HC subjects exposed to continuous lipid-lowering treatment than in naive patients (Perez-Calahorra et al., 2019). In another large study including patients with “definite FH” diagnosis previously treated with statins and non-personal history of IHD, cardiovascular risk was not greater than the general population after 20 years of follow-up. It underlines the clinical utility of identifying subjects with FH before they have developed IHD and ensuring they receive intensive lipid-lowering therapy (Humphries et al., 2018). In FH children from 10 years old and above, early treatment with moderate or high intensity statins has reported a reduction in LDL-C between 28 and 54% with no associated secondary effects (Ramaswami et al., 2020). The addition of ezetimibe to statins for the treatment of HC has been shown equally effective in decreasing LDL-C in FH children and adults (Kastelein et al., 2008; Kusters et al., 2015).

Moreover, a different response to high-intensity statins between FH and PH subjects has been observed. A higher response to statins was observed in subjects with no genetic diagnosis of FH than those with defective or null alleles, being the last ones whom the objective of LDL-C <100 mg/dl is less frequently achieved (47.4, 27.1, and 47.4%, respectively, *p* = 0.02) (Santos et al., 2014). More recently, in a study of treatment with high-intensity statins carried out in FH subjects, a significant reduction in LDL-C was observed in the p.(Leu167del) carriers on *APOE* (−52.1%) with respect to *LDLR* carriers (−39.7%) (*p* = 0.040) (Bea et al., 2019).

The decrease of LDL-C <100 mg/dl or at least 50% of the basal LDL-C concentration has been advocated by observational studies as the objective for primary prevention in populations with severe HC (Nordestgaard et al., 2013; Mach et al., 2020). However, this target is not attained for the greatest proportion of HC and FH populations (Perez-Calahorra et al., 2019; Pérez de Isla et al., 2019). Furthermore, <50% of these subjects, particularly young ones, are in chronic treatment with a high-intensity statin (Benn et al., 2012; Bucholz et al., 2018; Kotseva et al., 2019; Patel et al., 2019). The addition of other cardiovascular risk factors may modulate the intensity of the treatment in the rest of subjects with moderate HC (Mach et al., 2020). There is neither a minimum level of LDL-C below which benefit has not been observed nor has been observed a higher incidence of secondary effects associated with lower concentrations of LDL-C. PCSK9 inhibitors, the most potent lipid-lowering treatment available so far, reach an extra reduction of 50–60% on LDL-C with regard to conventional treatment. It has been proben to be effective to get LDL-C objectives in more than 50% of FH subjects (Kastelein et al., 2015; Santos et al., 2020), reducing the risk of cardiovascular events proportionally in the same grade than statins (Karatasakis et al., 2017; Giugliano et al., 2018) (Figure 1). Besides, PCSK9 inhibitors have proved to regress atherosclerotic plaques in coronary arteries with respect to conventional treatment in subjects with LDL-C ≤ 100 mg/dl (Nicholls et al., 2016; Bea et al., 2017). Nowadays, due to the high price of PSCK9 inhibitors, its use has only been established in FH subjects with a very high risk or personal history of ASCVD (Ascaso et al., 2019). Recent data suggest that only 23% of FH subjects in treatment with moderate- to high-intensity statins with or without ezetimibe reach LDL-C <100 mg/dl, whereas only 12% of those with a personal history of ASCVD had LDL-C <70 mg/dl. Nevertheless, it is estimated that only 17% of FH subjects may be eligible for treatment with PCSK9 inhibitors, according to current European guidelines (Masana et al., 2017).

## Discussion

In the last years, high-quality researches have been developed trying to find out new genes causing primary HC. However, no new genes other than *PCSK9* and *APOE* have been added to the list of those causing FH, accounting only for a small proportion of these subjects (Nordestgaard et al., 2013; Ghaleb et al., 2018). At the same time, a deeper knowledge about genetics related to lipid metabolism has allowed to detect the effect of small nucleotide variants related to LDL-C metabolism as a new pathogenic mechanism causing primary HC (Willer et al., 2013; Khera et al., 2016; Berberich and Hegele, 2019 (Figure 2). Despite the description of a polygenic trait (Willer et al., 2013), its effect has not been replicated homogeneously in a significant proportion of subjects with severe HC, and a consensus with respect to the diagnosis of PH has not been released so far.

There has been observed a higher cardiovascular risk in FH with respect to PH subjects, especially at early ages (Khera et al., 2016; Trinder et al., 2019). Moreover, the increased cardiovascular risk associated with PH is mostly observed in adulthood, when other cardiovascular risk factors occur in the same subject, underlining the effect of the environment or behavior on the phenotypic expression of genetic traits (Humphries et al., 2018; Perez-Calahorra et al., 2019; Trinder et al., 2020).

Despite the above facts, a large proportion of subjects with FH remains underdiagnosed and undertreated. New strategies are required to improve the diagnosis and treatment of HC subjects: cascade screening on first- and second-degree relatives of an FH proband or a more inclusive one by LDL-C measurement of first-degree relatives from any HC proband. Early diagnosis and treatment with moderate- to high-intensity statins result in the best approach to primary prevention disease in HC subjects, independently of the genetic background. Indeed, the use of high-intensity statins for 12 years allowed to avoid 90% of ASCVD in a large FH population (Perez-Calahorra et al., 2019). Only a small proportion of HC subjects as those with familial history of ASCVD or more than one risk factor associated may require a more aggressive treatment for primary prevention of ASCVD (Ascaso et al., 2019; Mach et al., 2020).

## Conclusion

Severe HC is a clinical condition that constitutes an important public health issue. Besides the genetic diagnosis of FH, new genetic methods have allowed to detect the additive effect of several variants in a unique nucleotide as a cause of PH. Nonetheless, a unique genetic criterion for the diagnosis of PH has not been established so far. Both FH and PH are associated with increased risk of ASCVD. The early diagnosis of severe HC in addition to a familial history of dyslipidemia and/or premature ASCVD is the starting point to detect those forms of HC more related to a genetic defect and with increased risk of ASCVD. Early treatment with high-intensity statins is mandatory for primary prevention of ASCVD in all subjects with severe HC.

## Author Contributions

EJ drafted the manuscript. IL-M, VM-B, and AB-S revised the manuscript. EJ, AB-S, and IL-M contributed to the acquisition and interpretation of data. EJ designed the work. All authors contributed to the manuscript revision, read, and approved the submitted version.

## Conflict of Interest

The authors declare that the research was conducted in the absence of any commercial or financial relationships that could be construed as a potential conflict of interest.

## Abbreviations

ASCVD: atherosclerotic cardiovascular disease
DLCN: Dutch Lipid Clinic Network
FH: familial hypercholesterolemia
GWAS: genome-wide association study
HC: hypercholesterolemia
IHD: ischemic heart disease
LDL-C: low-density lipoprotein cholesterol
non-HDL-C: non-HDL cholesterol
PH: polygenic hypercholesterolemia
TC: total cholesterol
TG: triglycerides
TX: tendinous xanthoma
SNV: single nucleotide variant.
